# New Insights on Retrieval-Induced and Ongoing Memory Consolidation: Lessons from Arc

**DOI:** 10.1155/2015/184083

**Published:** 2015-08-24

**Authors:** Jean-Pascal Morin, Kioko Guzmán-Ramos, Federico Bermudez-Rattoni

**Affiliations:** ^1^Instituto de Neurobiología, Universidad Nacional Autónoma de México, Campus Juriquilla, Boulevard Juriquilla 3001, Col. Juriquilla, 76230 Santiago de Querétaro, QRO, Mexico; ^2^Instituto de Fisiología Celular, UNAM, Ciudad Universitaria, 04510 México, DF, Mexico; ^3^Departamento de Ciencias de la Salud, Unidad Lerma, Universidad Autónoma Metropolitana (UAM), Avenida de las Garzas No. 10, 52005 Lerma, MEX, Mexico

## Abstract

The mainstream view on the neurobiological mechanisms underlying memory formation states that memory traces reside on the network of cells activated during initial acquisition that becomes active again upon retrieval (reactivation). These activation and reactivation processes have been called “conjunctive trace.” This process implies that singular molecular events must occur during acquisition, strengthening the connection between the implicated cells whose synchronous activity must underlie subsequent reactivations. The strongest experimental support for the conjunctive trace model comes from the study of immediate early genes such as c-fos, zif268, and activity-regulated cytoskeletal-associated protein. The expressions of these genes are reliably induced by behaviorally relevant neuronal activity and their products often play a central role in long-term memory formation. In this review, we propose that the peculiar characteristics of Arc protein, such as its optimal expression after ongoing experience or familiar behavior, together with its versatile and central functions in synaptic plasticity could explain how familiarization and recognition memories are stored and preserved in the mammalian brain.

## 1. Introduction: Characterization of IEGs and the Particularities of Arc

The immediate early genes (IEGs) were first described in viruses and then identified in various cell lines. The IEGs are transcribed following a variety of stimulations such as growth factors, hormones, and cytokines in a protein synthesis-independent fashion [1]. Their relevance for the study in adult neuronal plasticity was first brought to light in 1987, when it was shown that c-fos, a protooncogene that is also a transcription factor, was rapidly transcribed in neurons following seizures [2]. A couple of years later, another transcription factor, zif268, was identified; it was expressed after plasticity inducing treatments such as maximal electroconvulsive shocks and long-term potentiation (LTP). It has also been demonstrated that zif268 transcription is dependent on N-methyl-D-aspartate (NMDA) receptors activity, suggesting a functional link between these receptors and IEGs in the process of synaptic plasticity [3, 4]. In the following years, Paul Worley and collaborators undertook the task of identifying IEGs whose products were directly involved in modifying cellular function, rather than transcription factors with a presumably indirect role [5]. This gave rise to the discovery of a whole new set of “effector” IEGs: the COX-2 [6] an enzyme involved in lipid metabolism that was later shown to be involved in long-term plasticity and memory [7], Homer1a, a scaffold protein that interacts with metabotropic glutamatergic receptors and modulates intracellular calcium signaling [8], and activity-regulated cytoskeletal-associated protein (Arc), a protein involved in synaptic remodeling and plasticity [9–12]. These IEG products appeared as excellent candidates for proteins whose ongoing synthesis is essential for LTM to occur. However, an obvious intriguing question remained in how do proteins, newly synthesized in the soma, become associated with potentiated synapses?

In order to explain that question, the concept of “synaptic tagging” was introduced. Synaptic tagging is the idea that a translation-independent molecular mark must be established at potentiated synapses in order to provide input specificity for long-term, protein synthesis-dependent plasticity mechanisms [13, 14]. With Arc being a candidate for plasticity related proteins recruited by putative synaptic tags, its discovery was particularly encouraging for a number of reasons. After LTP-inducing stimulation of the perforant path, Arc mRNA was shown to accumulate specifically in the medial molecular layer of the dentate gyrus (DG), that is, the dendritic region that received the bulk of the stimulation during this procedure [15, 16]. Importantly, this phenomenon was later explained by the dendritic transport of its mRNA, which also was obliterated by NMDA receptors antagonism [15–18].

Further insight on the involvement of Arc in memory formation was gained when researchers examined the dynamics of Arc mRNA in the hippocampal network after exploration of a novel environment. That is, after 5 min of spatial exploration Arc mRNA was reliably detected in the nuclei of activated cells of the hippocampus and cortex. Interestingly, 25–30 minutes later, the percentage of cells expressing Arc mRNA in the nucleus was comparable to that of control animals, as the transcript already traveled to the cytoplasm where it was reliably detected [19, 20]. This kinetics of Arc mRNA combined with the specificity to physiologic stimuli [19, 21] has allowed the design of a method combining in situ hybridization and confocal microscopy to detect large neuronal populations activated by two or even three distinct behavioral epochs [22, 23]. This tool, termed catFISH (for “cellular compartment analysis of temporal activity by fluorescence in situ hybridization”), has helped to advance our understanding of the neuronal circuit underlying memory storage in a variety of behavioral paradigms. The catFISH technique allowed demonstrating that the population of cells expressing Arc during a subsequent exposure to the same environment highly overlaps with those expressing the mRNA during the first period. However, when the two behavioral epochs consisted in two strikingly distinct environments, the populations of cells expressing Arc were shown to be statistically independent. Noteworthy,* in vivo* single unit recordings have shown that, during exploratory behavior in rats, ~18% of CA3 and ~40% of CA1 neurons show “place field” activity. Interestingly, it was discovered that a similar proportion of neurons express Arc mRNA in the nucleus. Thus, since these place cells are widely believed to store contextually relevant information [24], this further pointed to a role in Arc in declarative memory that was consistent with the conjunctive trace model. Accordingly, it was demonstrated that acute intrahippocampal inhibition of Arc translation during the hours following acquisition impaired LTM of a spatial navigation task [25]. A more recent study by the same group showed that inactivation of the medial septum, a treatment known to impair hippocampus-dependent learning and memory [26], abolishes behaviorally induced Arc expression in this region [27]. Importantly medial septum inactivation is known to spare location specific firing in CA1 place cells [28]. These findings thus strongly suggest that Arc expressing neurons represent a memory storing engram rather than neuronal activity* per se* and further strengthen the rationale behind mapping Arc gene expression in neuronal networks during behavior.

Importantly, Arc expression mapping has been helpful to visualize memory storing neuronal networks not only in the hippocampus but also in several cortical and subcortical regions under a wide variety of behavioral paradigms. For example, some researchers took advantage of the conditioned taste aversion (CTA) task in which a strong associative memory is formed even if the conditioned stimulus (a novel taste) and the unconditioned stimulus (postingestive induced malaise) are presented 25 min or even more apart [34]. This considerable time lapse between stimuli allowed the authors to perform a catFISH design allowing visualization of the convergence of a conditioned stimulus with the unconditioned stimulus onto single neurons in the basolateral amygdala [35]. Indeed, some amygdala neurons were activated by both stimuli (had both nuclear and cytoplasmic Arc mRNA). However, when the stimuli presentation was reversed, that is, the LiCl injection was first and then the saccharin solution was presented after 25 min, the proportion of double stained amygdala neurons was dramatically decreased. These results strongly suggested that the observed convergence in the forward conditioning represented associative learning rather than mere overlap in the neuronal response [35]. Later, inspired by this study, another group of researchers used the conditioned odor preference task and showed that neurons of basolateral amygdala “learned” to associate an odor with an appetitive taste outcome, as a repeated convergence of taste and odor induced Arc mRNA increments after several days of pairing the smell with the taste [36]. A similar phenomenon was observed in the insular cortex by another group; they showed that an odor cue associated with a taste was as efficient at driving IEG expression in insular cortex neurons as the taste itself [37]. Moreover, in this study it was found that when the same taste was presented twice, it tended to induce IEG transcription (Arc and Homer1a) in the same subset of neurons in the insular cortex, just as it occurred in the hippocampus after repeated exploration of the same environment [37].

## 2. Molecular Mechanisms of Arc-Dependent Synaptic Plasticity

### 2.1. Tight Regulation of Arc Expression

As mentioned earlier, intranuclear foci of immature Arc can be detected 2 to 5 min after exposing rats to an open field [19]. If the groups of neurons that express Arc after information encoding were memory storing networks, one would expect that changes in synaptic activity would play a major role in this fast and discrete Arc expression. Efforts were thus deployed at identifying the precise cascade of events, from the synapse to the nucleus, that give rise to Arc expression. A role of putative memory-associated signaling pathways was early suspected and, accordingly, it was found that depolarization-induced Arc in neurons was dependent on intracellular calcium influx and activation of cAMP dependent protein kinase and extracellular signal regulated kinase signaling pathways [38]. Later, another group showed that glutamate release at excitatory synapses induces rapid Arc mRNA transcription in hippocampal neurons by a mechanism that depends on the transcription factor Myocyte Enhancer Factor type 2 activation [39]. However, the effects on Arc expression obtained in these studies were rather modest considering the robust increase observed under physiological conditions [16, 19, 27]. A more recent study further sought to identify highly preserved* cis*-acting elements in the Arc promoter that could account for the very tight and dramatic activity-dependent increase of Arc transcription reported in earlier studies. Screening more distal parts of the Arc promoter (~7 kb) they found a ~100 bp element that was sufficient to replicate the full extent of Arc's activity-dependent induction (~150 fold increase) after periods of intense activity* in vitro* and coined this element, “synaptic activity responsive element” (SARE). Importantly blocking *α*-amino-3-hydroxy-5-methyl-4-isoxazolepropionic acid (AMPA) and NMDA receptors abolished SARE-induced transcription [40]. Noteworthy, regions within the SARE element matched consensus binding sequences for cyclic adenosine monophosphate responding element binding protein, serum response factor, and Myocyte Enhancer Factor type 2 (CREB, SRF, and MEF2, resp.), three transcription factors strongly involved in neural plasticity [41]. Therefore, this element provided a mechanism by which Arc transcript can be strongly induced, specifically by synaptic activity.

In addition to the activation of the SARE element, a mechanism that ensures rapid synaptic activity-dependent Arc transcription was recently unveiled, which resides in stalled RNA polymerase II at the transcription initiation starts of Arc promoter [42]. Poised polymerase, along with active chromatin marks and preloaded transcription factors, provides a mechanism by which an activity induced signal can bypass the time-consuming process of transcription initiation and release RNA polymerase II for active transcription [42]. Interestingly, interfering with RNA polymerase stalling affected rapid induction of Arc but spared delayed IEG such as early growth response protein 3. Thus, these new findings on the molecular events that underlie Arc transcription help to explain how it can exert its function in behaviorally activated cells, in a fast and specific manner.

### 2.2. Arc Localization and Function

#### 2.2.1. Synaptic Strength Decrease

Experiments aimed at uncovering the role of Arc in synaptic plasticity at molecular and cellular levels showed that, in dendritic spines, Arc associates with the endocytic machinery, interacting with dynamin and endophilin 2/3, components of the clathrin-dependent endocytic machinery, thus enhancing AMPA receptors endocytosis [43]. Arc is strongly induced in neurons where its protein downregulates surface AMPA receptors after periods of increased neural activity. Thus activity induced Arc has a role in homeostatic synaptic scaling [44, 45], a non-Hebbian form of plasticity that serves to shift back neural excitability to physiological range, while preserving the relative change in individual synapses induced by Hebbian forms of plasticity, such as LTP [46–48]. Moreover, rapid dendritic translation of “constitutive” Arc mRNA has been shown to underlie metabotropic glutamate receptors- (mGluR-) dependent long-term depression (LTD) through Arc-dependent AMPA receptors endocytosis [49].

The role of Arc in the cell-wide weakening of glutamatergic synapses seemed counterintuitive, based on abundant evidence showing accumulation of both Arc mRNA and protein in potentiated dendritic regions [15, 29], as well as its requirement for LTP maintenance [25, 50]. However, groundbreaking new evidence was brought to light in a recent paper by Hiroyuki Okuno and collaborators that reconciled the role for Arc in synapse-specific homeostatic plasticity and synaptic tagging [51]. The authors first used a yeast two-hybrid screening to identify protein partners binding to Arc and identified an interaction with calcium/calmodulin-dependent protein kinase II *β* (CAMKII*β*). This interaction was found to be stronger in the absence of the Ca^2+^/CaM complex, suggesting a preferential interaction with the inactive form of the kinase. Moreover, after reliably and robustly inducing global Arc expression in neurons, the authors examined the effect of locally suppressing synaptic activity at single presynaptic sites. Strikingly, this treatment increased Arc accumulation at the inactivated synapses and, there, Arc was shown to diminish surface AMPA receptor GluR1 subunit content. Together, these results show that after periods of increased activity that induce robust Arc expression in neurons, it specifically accumulates at inactive spines by interacting with the inactive form of CAMKII*β*, that is not bound to calmodulin, enabling what was termed “inverse” synaptic tagging. The role of Arc, therefore, appears not only to scale down neural excitability after Hebbian synaptic modifications but also to crucially increase the contrast between potentiated and nonpotentiated synapses [52]. As mentioned previously, global Arc mRNA increments have consistently been observed at recently activated dendritic regions [22, 29]. It is probable that, under the settings used in these studies and as acknowledged by the authors, LTP occurs only in a subset of the stimulated synapses [29], as is also thought to occur during learning [53]. Conceivably, accumulation of Arc mRNA at activated dendrites or dendritic zones could provide a mechanism where inactive synapses in the vicinity of recently potentiated ones swiftly recruit massive amounts of Arc protein for synaptic depression to occur at these sites. However, as we shall see in the next section, a wealth of* in vivo* evidence also argues in favor of a distinct and specific role for* de novo* Arc translation in LTP consolidation at recently stimulated synapses.

#### 2.2.2. Synaptic Strength Increase

A growing body of evidence in favor of a direct role for Arc in synapse strengthening, at least under certain conditions, has recently received further support. First to be mentioned is that* de novo* Arc protein synthesis was soon shown to be required* in vivo* for the maintenance phase of LTP of the perforant path [25]. Later studies in Arc knockout mice confirmed a role for Arc in both LTD and LTP. Specifically, LTP induction was shown to be enhanced, while the maintenance phase was abolished in both perforant path and Schaffer collateral pathways, in agreement with the previous findings [50]. However, the strongest piece of evidence in favor of LTP consolidation appears to come from studies using perforant path stimulation of DG's granule cells. Noteworthy and contrary to what happens in hippocampal pyramidal cells where both LTP and novel environment exploration induce a robust and temporally discrete wave of Arc expression, a more gradual and sustained increase of Arc mRNA and protein appear to be produced by these procedures in DG's granule cells [33, 54–56]. Importantly, local infusions of Arc asODNs at 2 h following* in vivo* high frequency stimulation of the perforant path abolished LTP maintenance and impaired F-actin polymerization and cofilin phosphorylation, molecular events that are thought to underlie learning-induced structural plasticity [30, 55, 57]. Most strikingly, treatment with the actin stabilizing drug jasplakinolide, between LTP induction and Arc asODNs treatment, abolished the deleterious effects of Arc translation inhibition on LTP maintenance. These results strongly suggest that Arc's role in DG LTP consolidation rests in its ability to stabilize recently polymerized actin filaments [55]. Finally, Arc asODNs infusions before LTP induction with high frequency stimulation or BDNF infusions prevented LTP expression indicating that Arc translation was required for early LTP expression as well as maintenance.

Recently mechanistically distinct rounds of translation that depended on sustained MNK activation through BDNF signaling were shown to underlie DG-LTP [58]. Infusions of BDNF scavenger TrkB-Fc or MNK inhibition brought field evoked postsynaptic potentials as well as Arc protein translation back to baseline. All in all, a very strong case can now be made for a direct role of Arc in DG-LTP. As observed before [59, 60] this quite strikingly contrasts with Arc's role in glutamatergic synapses weakening. However, nothing supports the* a priori* principle that Arc's function should be similar in every studied cell type. In fact, its role may differ between pyramidal and granule cells, as it was recently proposed for BDNF's [61]. This possibility should draw serious attention given that, as mentioned earlier, it is now demonstrated that Arc expression kinetics in granular and pyramidal cells differ dramatically. Further, still little attention has been paid to possible posttranslational modifications to Arc protein as it was observed in an earlier paper [59]. However, possible phosphorylation sites for PKC and CamKII have been identified since the protein's discovery [9]. As pointed out recently, and regardless of the experimental settings or cell type, the bulk of Arc protein observed in principal activated cell appears in the perinuclear cytoplasm, where its function remains obscure [60] although it is now established that at least part of it is shuttled to the nucleus.

#### 2.2.3. Arc in the Nucleus: Cell-Wide Homeostatic Downscaling of AMPA Receptors

Arc protein was first detected in the cell nucleus of cultured hippocampal neurons in association with promyelocytic leukemia bodies (PML), which are putative sites of transcriptional regulation [62]. Consistently, a more recent study further showed that stimulating DG granular cells for prolonged periods, with brain-derived neurotrophic factor or bicuculline, induced a gradual targeting of Arc to the nucleus that reaches peak levels at 8 h. There, Arc promotes the assembly of nuclear PML bodies, which, in turn, negatively regulate the transcription of the AMPA receptor subunit GluR1. Importantly also, nuclear localization was also observed after exposure to a novel environment not only in the granular cells of the DG but also in hippocampal CA1 and CA3 regions and in the somatosensory cortex. Importantly, the kinetics of Arc accumulation to the nucleus varied depending on the brain region and cell type. These findings thus provide an additional, cell-wide mechanism by which Arc promotes homeostatic plasticity, after prolonged periods of synaptic activity [33]. All in all, Arc accomplishes distinct functions depending on its interaction partners and the time course of its accumulation.

### 2.3. Spine Type-Specific Accumulation of Arc Protein

**Figure 1:**
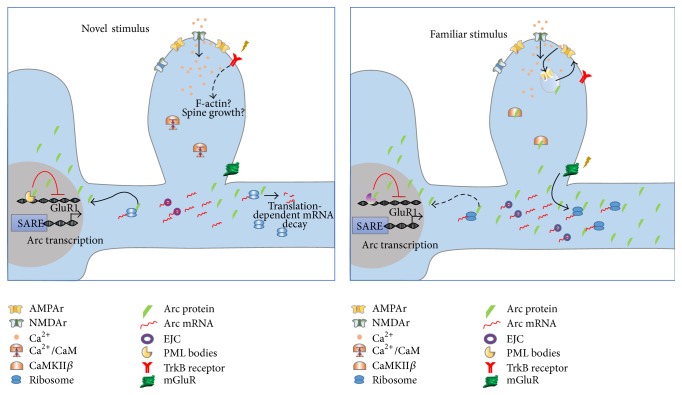
Hypothetical model of the differential role of Arc expression after the presentation of a novel and a familiar stimulus. In both cases, active Arc expressing cells are presented in which a swift and massive calcium entry through NMDA receptors at synaptic sites induce dramatic increase in Arc mRNA expression through “SARE” activation. Further NMDA receptors activation and increased protein synthesis observed after novelty exposure could induce synaptic activity and translation-dependent Arc mRNA degradation as it was observed after DG-LTP [29]. In the novelty condition, increased TrkB activation through BDNF may lead to increase in actin polymerization and spine growth at potentiated synapses, mechanisms that, in addition to LTP, are thought to underlie the consolidation of novel information [30]. On the other hand, familiarization primes dendrites for mGluR1-LTD and increased Arc protein synthesis [31, 32] arguably after reactivation of the same circuit. In addition to synapse-specific downregulation of surface AMPA receptors, a more global, cell-wide mechanism occurs in which Arc is shuttled to the nucleus and associates with PML bodies to repress GluR1 transcription. Accumulation of Arc in the nucleus has been observed in the hours following novel environment exploration [33] and may also occur after familiar stimulus exposure.

Another interesting observation is that the Arc-dependent downregulation of surface AMPA receptors appears to be specific to certain dendritic spines, depending on their morphological characteristics. Dendritic spines can indeed be classified in distinct categories according to their shape, size, and structure, which are correlated with synaptic strength, motility, and structural plasticity. “Mushroom” spines are larger, are much more stable, and have a greater amount of AMPA receptors than “thin” spines that are also much more labile and dynamic. For these reasons, mushroom spines have been referred to as “memory spines,” whereas thin spines are the putative “learning spines” [63]. In agreement, thin spines are more susceptible to Arc-dependent GluR1 endocytosis [64]. Further, Arc knockout mice have increased seizure sensitivity and epileptiform activity as measured with electroencephalogram, whereas Arc −/− neurons have decreased spine density but, crucially, increased spine width [64]. These findings confirmed a role for Arc in homeostatic synaptic scaling and global network stability. Arc protein targeting at synapses “tagged” as inactive would diminish unspecific noise and allow nearby potentiated Arc-negative thin spines to stand out and eventually become “memory spines.” Conceivably, synaptic potentiation and spine growth could be the “default” mechanism that occurs in behaviorally activated cells; it well could be that synaptic inactivity could be the trigger that confers specificity. Alternatively, distinct, yet complementary, mechanisms could occur at active synapse that would further increase the contrast between potentiated and unpotentiated synaptic networks (see Figure 1).

## 3. The Requirement of Arc for LTM Formation

Given the synaptic localization of Arc protein, its tight activity dependence, and its striking effects on synaptic function, efforts have been deployed to uncover its possible role at distinct phases of the process of learning and memory. The generation of Arc knockout mice revealed a role for Arc in long-term but not short-term memory in a variety of tasks, including object recognition memory and amygdala-dependent tasks, such as conditioned taste aversion and fear conditioning, showing that learning* per se* is unaffected in these mice [50]. Furthermore, the formation of long-term spatial memory as assessed by Morris Water Maze task was impaired; Arc knockout mice were slower learners, formed a less precise memory, and, interestingly, showed less behavioral flexibility as they took longer to relearn a new position of the target platform [50]. These results in the Arc knockout mice were in accordance with the seminal paper of Guzowski and collaborators mentioned earlier that found impaired long-term spatial memory after acute Arc translation blocking, therefore providing a first causal link between* de novo* Arc protein synthesis and LTM consolidation [25]. A requirement for* de novo* Arc protein expression for consolidation and reconsolidation processes was later unveiled in a great variety of learning and memory paradigms. For example, in the amygdala, a key structure involved in the storage of the emotional contingency related to a context or a stimulus [65, 66], it was shown that administration of Arc antisense oligodeoxynucleotides (asODNs) before training the animals in a Pavlovian fear conditioning task affects its consolidation [67]. Furthermore Arc asODNs administration 90 min before reactivation of the same task in the lateral amygdala impaired reconsolidation of this task [68]. On the other hand, infusions of Arc asODN in the basolateral amygdala 3 h before extinction of a contextual fear conditioning task impaired shifting of the emotional component of the context from aversive to safe [69].

Similarly, the importance of* de novo* Arc translation for LTM formation was also demonstrated in the neocortex, in both associative and nonassociative memory paradigms. For example, posttraining administration of Arc asODNs in the cingulate cortex was reported to disrupt LTM formation in an inhibitory avoidance paradigm [70]. In our lab, we showed that inhibiting Arc protein synthesis in the insular cortex prevents familiarization with a safe taste and hinders the hedonic shifting of a taste from aversive to safe during extinction of conditioned taste aversion (CTA) (Guzman-Ramos et al., manuscript in preparation). Taken together, these data demonstrate that* de novo* Arc protein expression in critical mammalian forebrain structures plays an essential role in LTM formation. Therefore, while some factors have been identified that specifically operate in either consolidation or reconsolidation, this does not seem to be the case of Arc which synthesis appears to be required indistinctively for both processes [71, 72]. These studies indicate that* de novo* Arc protein synthesis in the participating brain structures seems to be required for both processes. Furthermore, they are in agreement with observations by ours and other groups showing that Arc protein expression increased once a behaviorally relevant stimulus becomes familiar (see below).

### 3.1. Is Synergy of Arc-NMDA Receptors Necessary for LTM?

In many tasks, the requirement for NMDA receptors activity in order to consolidate LTM has been clearly established [73]. A functional link between NMDA receptors activation and IEGs expression has long been suspected to underlie specific synaptic modifications that are essential for the establishment of a stable memory trace. As a matter of fact, inhibition of NMDA receptors is known to hinder activity-dependent Arc mRNA expression, localization at activated dendritic sites, and degradation [9, 15, 29]. However, a direct involvement of Arc-NMDAR interdependency* in vivo* during learning was not tested until recently. To this matter, one group used contextual fear conditioning, a task known to involve NMDA receptors-dependent plasticity mechanisms in the hippocampus [74], and showed an increase in Arc protein accumulation in the hippocampus at 1 h after acquisition that was blocked by NMDA receptor antagonist APV. Furthermore, pretraining infusions of Arc asODNs impaired consolidation but spared acquisition of contextual fear conditioning tasks [75]. These results clearly point to a role of NMDA receptors-dependent Arc synthesis in the hippocampus in the formation of a long-term contextual fear memory. Yet a more recent study sought to determine whether this Arc-NMDA receptors synergy was also involved in memory retrieval of a familiar task. Moreover, NMDA receptors activity upon retrieval proved to be essential for contextual memory maintenance as the increased locomotion upon subsequent context exposure was attenuated in rats treated with NMDA receptors antagonist APV [76]. These findings bring to light further support for the idea that retrieval memories, even well-consolidated ones, place the involved circuits in a labile state that require further NMDA receptors-dependent Arc protein synthesis for their stabilization. In the next section we will discuss recent findings regarding the phenomenon of retrieval-induced plasticity mechanisms, with a focus on Arc, and their possible role in memory stabilization and persistence.

## 4. Memory Circuits Reactivations and Ongoing Synaptic Plasticity

Hebb's second postulate stipulated that, in addition to feed-forward synaptic strengthening, reverberation of neural ensembles must occur in order to form a temporary unit of memory storage. These ensembles of neurons facilitate coincidence detection of upcoming sensorial information by integrating information from temporally related, but spatially segregated, neural activity [77]. In recent years, several lines of experimental evidence have brought support and refined Hebb's proposal. In our lab, we examined putative offline reactivations after associative learning at the level of neurochemical extracellular changes, using the conditioned taste aversion paradigm. First, we found a significant increase of dopamine levels in the insular cortex during the ingestion of a novel saccharin solution. Second, intraperitoneal injection of LiCl, used as an unconditioned stimulus in this task, was shown by itself to produce a swift increment of glutamate release in this same structure. Strikingly, however, a delayed, concomitant release of dopamine and glutamate was observed in the insular cortex that was abolished by reversible inactivation of the amygdala, another structure involved in long-term conditioned taste aversion memory formation [78, 79] (interestingly, similar results with the neurotransmitters norepinephrine and glutamate were observed in the amygdala [80]). Arguably, the concomitant dopamine and glutamate release we reported in the insular cortex could precede and be required for more enduring forms of synaptic plasticity in these regions that were reported by our group and others [37, 81, 82].

### 4.1. Epigenetic Modulation of Arc Expression

Currently, the most widely accepted model accounting for LTM formation stipulates that learning induces morphological and functional modifications at activated synapses and subsequent learning-dependent protein synthesis allowing stabilization of these modifications, so that the newly strengthened synaptic networks become stored for days to months, a phenomenon that was termed synaptic consolidation [83]. Recently, an emerging subfield of neuroepigenetics, the study of the role of epigenetics mechanisms in adult neurons [84], has recently unveiled possible mechanisms by which synapse-specific changes induced by learning could remain permanently. In this regard, the molecular mechanisms of memory maintenance focused on the cell's nucleus have been proposed; that is, covalent modifications of the DNA are the ultimate biochemical event that could store information permanently [85]. Notably, this phenomenon could work in parallel with wider distribution of the memory trace through cortical networks in order to further stabilize memories. The concept of epigenetics refers to “changes in gene transcription through modulation of chromatin, which are not brought by changes in DNA sequence” [86]. There are two possible ways in which these modulations of chromatin could play a role in memory storage. On one hand, stable chromatin modifications can interdigitate with synaptic tags in order to participate in and maintain synapse-specific changes [87]. Another possibility could be that neuroepigenetic mechanisms since they operate at a cell-wide level could induce metaplasticity in selected populations of neurons so that the tuning up or down of specific synapses would be permanently facilitated [87]. Importantly, epigenetic modifications at promoter sites of various plasticity related proteins, including Arc, have recently been described [88, 89].

As mentioned earlier Arc-dependent AMPA receptors endocytosis operates both at a synapses specific level, through synaptic inactivity-dependent interaction with CAMKII*β* [51, 52], and in a cell-wide fashion, through downregulation of GluR1 transcription [33]. Interestingly, methylation of the Arc promoter that correlated in time with a decrease in Arc protein below basal levels has been reported at 24 h after the induction of electroconvulsive seizures [90]. Also, aberrant changes in Arc promoter methylation in hippocampal neurons have been suggested to play a role in age-related cognitive decline [91]. Methylation, a putative gene silencing signal, is arguably the most stable epigenetic modification and could serve to maintain changes in gene expression dynamics induced by memory consolidation [92]. Indeed methylation of memory suppressing gene* calcineurin* was induced in the frontal cortex after contextual fear conditioning and persisted for at least 30 days. Further, interfering with the enzymes responsible for maintaining methylation on cytosine residues, on the 30th day after conditioning, significantly impaired retention of the task [92]. On the other hand, knocking-down of Tet1, an enzyme that promotes DNA demethylation, is associated with decreased expression of synaptic plasticity related, putative memory enhancer genes Nasp4, c-fos, Egr2, and Arc as well as abnormally enhanced LTD and impaired memory extinction [93]. This suggests that the methylation rate of these genes affects the effectiveness of subsequent plasticity inducing events, thus modulating their ability to update consolidated memories upon reactivation. Taken together, these findings provide crucial insights on the role of chromatin modifications in long-term memory persistence and will undoubtedly set the basis for important new discoveries in this field, in the years to come. Meanwhile, they might also provide a “rationale” behind the robust Arc and other plasticity related proteins' expression that is observed after retrieval of even “well-consolidated” memories, as well as during offline rest periods.

## 5. Familiar/Consolidated Tasks Induce Robust Arc Expression: Abundant Evidence but Still Elusive Function

Some characteristics of Arc expression during ongoing behavioral experience, especially at the posttranscriptional level, are somewhat counterintuitive, given its role in memory consolidation. In fact under many setups it has been observed that Arc mRNA and protein are still expressed at high levels after the animal experiences an already familiarized context or stimulus. Further, under some circumstances, exposure to a familiar behavioral stimulation induces even greater Arc expression. In a recent study exploring Arc mRNA expression dynamics in hippocampal subfields after running around a track in a novel context, optimal Arc expression was observed in CA3 after a rat ran around a track a single time; no further increment was observed when the animal ran several times around the same track or when it ran several times for four consecutive days. In CA1, on the other hand, the greater proportion of Arc expressing cells was observed in the condition where the animal ran several times around the track for the fourth consecutive day in the same context [27]. First of all, these data provided compelling support at the molecular level for a role of CA3 in the fast encoding and subsequent storage of a novel context. Further, it provided evidence that behaviorally induced plasticity mechanisms are still required in the hippocampus even when the environment is familiar [27] and is consistent with earlier studies that found robust spatial exploration-induced Arc mRNA expression in DG granular cells even after the environment was experienced for the ninth time [94]. Also in agreement with these findings, it was reported by the same group that Arc transcription in rats trained in the Morris Water Maze task is similar after overtraining compared to after initial acquisition [95]. All these findings suggest that active spatial exploration induce Arc-dependent plasticity mechanisms in the hippocampus every time it is reinstated, regardless of familiarity [95].

Strong evidence links LTD, that is, a decrease in the efficiency of synaptic communication, with the formation of object recognition memory, and recent evidence points to a role for Arc for this type of recognition memory. For example, exploration of a novel object has been associated with induction of LTD in CA1 network, since novel object exploration during low-frequency stimulation of the Schaffer-collateral pathway facilitated LTD in rats [31, 96]. Recently, it was shown that exposure to a novel environment induced strong dendritic expression of Arc mRNA in hippocampal CA1 pyramidal neurons, but translation remained tightly repressed. However, further exposure to the same environment lifted the break on Arc translation in the dendrite and allowed AMPA receptor-dependent LTD to proceed [97]. Therefore, an attractive possibility suggested in the same study could be that recognition memory operates in such a way that novelty primes activated neurons for LTD but Arc translation remains temporally suppressed, until subsequent experiences with the familiarized stimulus trigger Arc translation locally at the dendrite and, therefore, promote long-lasting depression, allowing a sparser memory trace to be established.

Parallel lines of evidence suggest that a similar mechanism could be involved under different sensorial modalities. Seeking to elucidate Arc expression dynamics in neocortical networks, a recent work analyzed how a previous exposure to a sound affects Arc mRNA expression in the auditory cortex after presentation of the same sound on the following day. The same proportion of neurons expressed Arc mRNA after rats were presented with the sound, whether it was novel or familiar. However, they detected a greater proportion of cells with Arc transcript in the cytoplasm specifically after exposure to the familiar sound [32]. These results provide compelling evidence that a single exposure to a noncontingent stimulus could modulate Arc expression dynamics in cortical networks and provide further evidence that Arc-dependent plasticity mechanisms are still occurring during behavioral familiarity. In our lab, we sought to evaluate Arc protein expression dynamics during taste recognition memory formation. We unexpectedly found that familiar saccharin consumption induced higher Arc protein accumulation in the insular cortex than novel saccharin, even when the amount of fluid consumed remained constant between the two conditions. Strikingly, local infusion of anisomycin in the dorsal hippocampus, a treatment known to affect taste familiarization [79], prevented the increase of Arc protein in the insular cortex observed on the second day. Further, immunofluorescence analysis revealed that the greater presence of Arc in the familiar condition was due to a dramatic increase in dendritic accumulation of the protein and that the same proportion of cells expressed Arc after both novel and familiar taste [98].

The fact that high levels of Arc protein expression are still observed even after the execution of a familiarized task could well be explained by memory consolidation-induced epigenetic changes that promote a shift in the transcriptional response of a given circuit upon subsequent reactivations. Also, it has been proposed that this sustained Arc expression after ongoing experience could serve to maintain the trace in a labile state in order to enable subsequent updating of the memory trace [27]. Consistently, extinction of an* in vitro* classical conditioning task induces similar synaptic levels of Arc protein more than initial acquisition does [99]. Moreover, recent findings from our lab have shown that* de novo* Arc protein synthesis in the insular cortex upon aversive taste memory retrieval is essential for aversive-to-positive hedonic shift of the taste valence (Guzmán-Ramos, Venkataraman, Morin, and Bermúdez-Rattoni, manuscript in preparation). However, the key question is whether Arc synthesis is required in asymptotically learned tasks, when no additional information or further updating is involved. Experiments from our lab found that optimal dendritic Arc protein expression occurred when the taste was presented for the fifth time, that is, when behavioral assessment of taste familiarization (Attenuation of Neophobia [34]) suggests that it is indeed asymptotically familiarized. Further, as mentioned earlier, other groups have found that Arc protein expression occurs in a similar proportion of cells after exploration of a novel and a familiarized environment [95]. These results indicate that* de novo* Arc protein is required every time a familiarization memory is reactivated, no matter how consolidated it is; however, no clear loss of function study has addressed this issue. Furthermore, most of Arc knockdown experiments have used asODNs, which only inhibit a fraction of Arc translation and are relatively unstable and subject to degradation mitigating their effects. The recent development of more stable asODNs, as well as* in vivo* virus-mediated knockdown experiments, could help address this question with more precision.

Finally, in addition to plasticity mechanisms induced by memory reactivation, ongoing synaptic modifications must occur offline in order to keep the memory trace stable. In keeping with this, it was discovered that offline wave of Arc protein expression in hippocampal networks occurred at 8 h and 24 h after spatial exploration [56]. In the DG, on the other hand, a single 5 min spatial exploration task was shown to produce sustained transcription of Arc mRNA in granular cells for as long as 8 hours [54]. Furthermore, it was shown that “basal” Arc mRNA expression in CA1 neurons during rest periods is not random but rather recapitulates previous experiences [100]. Importantly, it was shown that the fraction of neurons expressing Arc after spatial exploration and expressing it again during a subsequent rest period is highest in CA3 and lowest in the cortex [101], which is in accordance with the systems consolidation theory.

Here again, as a possible explanation for these so-called offline genomic reactivations, an epigenetic event, such as DNA methylation, could occur during initial acquisition and serve as an indelible footprint that allows for a subsequent round of synapse-specific consolidation to be accomplished every time the same network is solicited. Such an epigenetic tag could also alter the rate or susceptibility of transcription of certain genes in an ongoing fashion, in the absence of stimuli. In the future, more extensive characterization of learning-induced epigenetic modifications of Arc and other memory-related genes at specific loci will in our view greatly refine our understanding of how memories are dynamically stored and maintained over the range of years.

## 6. Conclusions and Further Issues

This review examined new findings on the role of Arc in long-term synaptic plasticity and memory formation. As we have seen, Arc's unique role in altering network function, possibly as a synaptic contrast enhancer, is reflected by the wide range of brain structures and memory paradigms in which its synthesis is required for LTM formation to proceed. Also particularly intriguing are the several models in which its translation is optimally promoted by stimulus familiarity rather than novelty or retrieval of a consolidated memory rather than establishment of a novel one. Further information on Arc's regulation mechanisms, particularly at the epigenetic level and on its molecular partners at the synapse should provide helpful insights for the emerging field of the neurobiological basis of memory persistence.
